# Ubiquitin-elongating enzyme Ufd2 confers resistance to hygromycin B in *Saccharomyces cerevisiae*

**DOI:** 10.17912/micropub.biology.002152

**Published:** 2026-06-30

**Authors:** Kaikeyi M Paxton, James A Avaala, Chance S Creviston, Joseph Gumina, Jason D True, Eric M Rubenstein

**Affiliations:** 1 Department of Biology, Ball State University; 2 Chicago College of Osteopathic Medicine, Midwestern University

## Abstract

Aberrant proteins are targeted for proteasomal degradation by polyubiquitylation catalyzed by sequential action of ubiquitin-activating enzymes (E1s), ubiquitin-conjugating enzymes (E2s), and ubiquitin ligases (E3s). A subset of proteasomal substrates require ubiquitin chain extension by ubiquitin-elongating enzymes (E4s) prior to proteolysis. We tested the requirement of Ufd2, the founding member of the E4 enzyme family, in resisting proteotoxic stress caused by the aminoglycoside hygromycin B in *Saccharomyces cerevisiae* . The human homolog, UBE4B, is a potential therapeutic target for neurological disease and cancer. *UFD2* deletion sensitized yeast to hygromycin B, consistent with a role for the E4 in protein quality control.

**Figure 1. UFD2 confers resistance to hygromycin B:** **(A)** The E3s Hrd1 and Doa10 mediate protein turnover at the endoplasmic reticulum (ER) via ER-associated degradation (ERAD). Hrd1 functions with the E2 Ubc7, which is tethered to the ER by Cue1. Doa10 works with two E2s, Ubc6 and Ubc7. Following Cdc48-dependent extraction from the ER, the E4 Ufd2 extends the polyubiquitin chains of a subset of ERAD substrates to accelerate proteasomal degradation. **(B, C)** Sixfold serial dilutions of yeast of the indicated genotypes were pipetted on medium lacking or containing hygromycin B. Plates were incubated at 30°C and imaged at the indicated times. Strains used in the experiment in **(B)** are derived from the BY4741 genetic background, whereas strains in **(C)** are derived from MHY500. Experiments were performed three or more times.

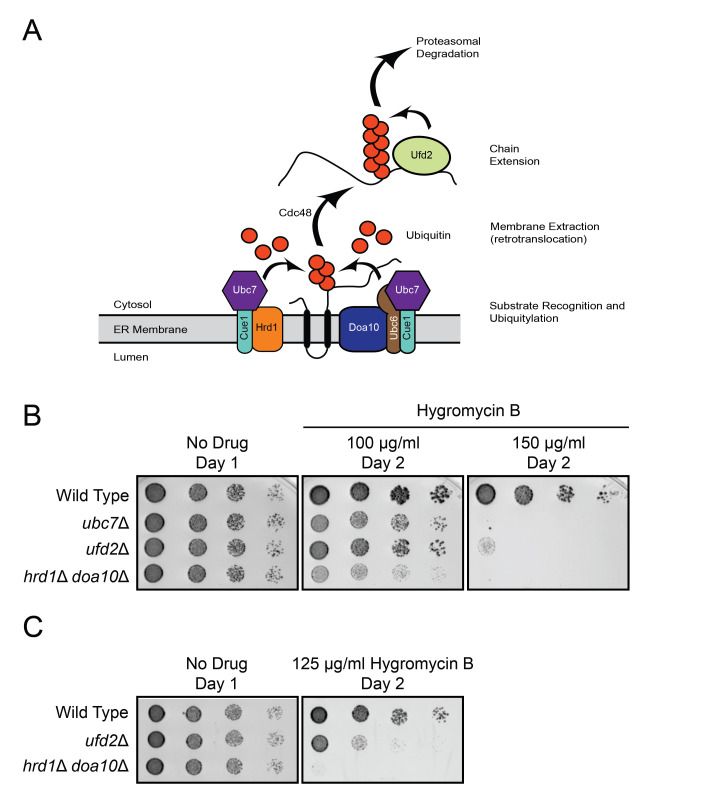



## Description

Protein quality control and regulated protein degradation through the ubiquitin-proteasome system (UPS) are required for cellular and organismal homeostasis. Through the sequential activity of ubiquitin-activating enzymes (E1s), ubiquitin-conjugating enzymes (E2s), and ubiquitin ligases (E3s), polymers of the small protein ubiquitin are covalently linked to proteins destined for proteasomal destruction (Kleiger & Mayor, 2014). In some cases, further elaboration of poly-ubiquitin chains by ubiquitin-elongating enzymes (E4s) accelerates protein degradation (Muller & Hoppe, 2024).

The *Saccharomyces cerevisiae* protein Ufd2 is the founding member of the E4 family of enzymes. It is structurally similar to the Really Interesting New Gene (RING) class of ubiquitin ligases (Koegl et al., 1999). Ufd2 contributes broadly to protein degradation, promoting turnover mediated by endoplasmic reticulum (ER)-associated degradation (ERAD) E3s ( Figure 1A ), the anaphase-promoting complex (APC), Skp1-Cullin-F-Box (SCF) enzymes, and other E3s (Anton et al., 2023; Liu et al., 2011; Liu et al., 2010; Nakatsukasa et al., 2008; Richly et al., 2005; Smith et al., 2016). Consistent with its broad substrate range, Ufd2 regulates homeostasis in multiple compartments, including the ER and mitochondria (Altin et al., 2025; Liu et al., 2010; Richly et al., 2005). Loss of Ufd2 sensitizes yeast to heat, ethanol, and cadmium stress in the context of dampened proteasome function caused by deletion of *RPN10* , which encodes a 19S regulatory particle subunit (Koegl et al., 1999).

UBE4B, the human homolog of Ufd2, is implicated in neurological health and tumor development. UBE4B plays a potentially protective role in the neurological disorder Machado-Joseph disease, as it promotes turnover of polyglutamine-expanded, pathogenic forms of ataxin-3 (Matsumoto et al., 2004). By contrast, UBE4B constitutively targets Charcot-Marie-Tooth disease variants of mitofusin, likely contributing to dysregulated mitochondrial dynamics and symptom progression in afflicted patients (Anton et al., 2023). Moreover, UBE4B also facilitates Mdm2-dependent degradation of the tumor suppressor p53 and inhibits tumor cell apoptosis (Wu et al., 2011). Thus, strategies that modulate UBE4B activity may be therapeutic or detrimental, depending on the condition.

Hygromycin B, an aminoglycoside produced by *Streptomyces hygroscopicus* , distorts ribosome A sites, resulting in mistranslation and impaired ribosomal translocation (Cabanas et al., 1978), likely increasing the abundance of aberrant proteins. We and others have demonstrated that mutations in several genes with demonstrated or predicted roles in protein quality control cause hygromycin B sensitivity (Akoto et al., 2025; Bengtson & Joazeiro, 2010; Chuang & Madura, 2005; Daraghmi et al., 2023; Flagg et al., 2023; Jaeger et al., 2018; Niekamp et al., 2019; Verma et al., 2013). For example, loss of genes encoding ERAD enzymes causes a profound growth defect in the presence of hygromycin B (Avaala et al., 2026; Crowder et al., 2015; Doss et al., 2023; Runnebohm et al., 2020; Turk et al., 2023; Woodruff et al., 2021).

Given its requirement for degradation of select ERAD substrates, we hypothesized that Ufd2 confers resistance to proteotoxic stress, such as that caused by hygromycin B. We assessed the fitness of wild type yeast, yeast lacking Ufd2, and yeast lacking ERAD components (either the ERAD E2, Ubc7, or two major ERAD E3s, Hrd1 and Doa10 (Mehrtash & Hochstrasser, 2019)) in the presence of sublethal concentrations of hygromycin B ( Figure 1B ). As in previous reports, deletion of *UBC7* or of *HRD1* and *DOA10* substantially impeded growth when hygromycin B was included in the growth medium (Crowder et al., 2015; Owutey et al., 2024). Indeed, loss of Ufd2 caused a marked growth defect in the presence of hygromycin B. Growth impairment in *ufd2* Δ yeast was not as severe as that observed in *ubc7* Δ and *hrd1* Δ *doa10* Δ yeast. Hygromycin B sensitivity of *ufd2* Δ yeast derived from a distinct genetic background further validates a role for this E4 in combatting proteotoxic stress ( Figure 1C ).

Our finding that *ufd2* Δ yeast are hypersensitive to hygromycin B is consistent with a general role for this E4 in protein quality control. Although plasmid-based complementation of *ufd2* Δ yeast would provide additional confirmation, reproducible observation of hygromycin B sensitivity in independently generated *ufd2* Δ strains from distinct genetic backgrounds (BY4741 (Brachmann et al., 1998) in Figure 1B and MHY500 (Chen et al., 1993) in Figure 1C ) increases confidence in this result. Future experiments will extend these analyses to define the spectrum of global and organelle-specific protein homeostasis stressors to which *UFD2* confers resistance. Hygromycin B sensitivity is not a general feature of E4 enzymes, as we previously observed that deletion of the gene encoding E4 Hul5 does not sensitize yeast to the drug (Woodruff et al., 2021). Together, these findings support an important role for Ufd2 in maintaining protein homeostasis and suggest that any future therapeutic strategies targeting the human homolog UBE4B should consider the potential consequences for protein quality control.

## Methods

**Growth assays**

Serial sixfold dilutions of *S. cerevisiae* were pipetted onto agar plates with yeast extract-peptone-dextrose (YPD) medium (Guthrie & Fink, 2004) lacking or containing hygromycin B (Gibco), as described in (Watts et al., 2015). Plates were incubated at 30°C. Hygromycin B concentrations were selected empirically to produce sublethal growth inhibition that permitted discrimination between strain fitness.

## Reagents

**Yeast strains used in this study.**

**Table:** 

**Name**	**Genotype**	**Figure**	**Reference**
**VJY6 (alias MHY500)**	*MATa his3-* Δ *200 leu2-3,112 ura3-52 lys2-801 trp1-1 gal2*	1C	(Chen et al., 1993)
**VJY8 (alias MHY1702)**	*MATa his3-* Δ *200 leu2-3,112 ura3-52 lys2-801 trp1-1 gal2 doa10* Δ *::HIS3 hrd1* Δ *::LEU2*	1C	(Huyer et al., 2004)
**VJY305 (aka SKY242)**	*MATa his3* Δ *1 leu2* Δ *0 met15* Δ *0 ura3* Δ *0 doa10* Δ *::kanMX4 hrd1* Δ *::kanMX4*	1B	(Habeck et al., 2015)
**VJY476 (alias BY4741)**	*MATa his3* Δ *1 leu2* Δ *0 met15* Δ *0 ura3* Δ *0*	1B	(Tong et al., 2001)
**VJY645**	*MATa his3* Δ *1 leu2* Δ *0 met15* Δ *0 ura3* Δ *0 ufd2* Δ *::kanMX4*	1B	(Tong et al., 2001)
**VJY1075**	*MATa his3* Δ *1 leu2* Δ *0 met15* Δ *0 ura3* Δ *0 ubc7* Δ *::kanMX4*	1B	(Tong et al., 2001)
**VJY1249 (alias MHY9433)**	*MATa his3-* Δ *200 leu2-3,112 ura3-52 lys2-801 trp1-1 gal2 ufd2* Δ *::kanMX6*	1C	Gift of Mark Hochstrasser and Adrian Mehrtash

## References

[R1] Akoto E, Doss EM, Claypool KP, Owutey SL, Richards KA, Lehman KM, Daraghmi MM, Turk SM, Indovina CJ, Avaala JA, Evans MD, Scott AR, Schneider HO, Rogers EM, True JD, Smaldino PJ, Rubenstein EM (2025). The kinesin Kar3 is required for endoplasmic reticulum-associated degradation.. Mol Biol Cell.

[R2] Altin S, Simões T, Behrendt C, Anton V, Domke D, Völtzke KM, Kumar R, Nolte H, Hermanns T, Brocke-Ahmadinejad N, Bendrin K, Zimmermann M, Büttner R, Ventura N, Krüger M, Dohmen RJ, Hofmann K, Hoppe T, Reichert AS, Escobar-Henriques M (2025). Ubiquitin precursor with C-terminal extension promotes proteostasis and longevity.. Mol Cell.

[R3] Anton V, Buntenbroich I, Simões T, Joaquim M, Müller L, Buettner R, Odenthal M, Hoppe T, Escobar-Henriques M (2023). E4 ubiquitin ligase promotes mitofusin turnover and mitochondrial stress response.. Mol Cell.

[R4] Avaala JA, Palackel M, Tesch IM, Gumina J, Creviston CS, True JD, Crowder JJ, Rubenstein EM (2026). Ubiquitin-conjugating enzyme membrane anchor Cue1 confers resistance to hygromycin B in Saccharomyces cerevisiae.. MicroPubl Biol.

[R5] Bengtson MH, Joazeiro CA (2010). Role of a ribosome-associated E3 ubiquitin ligase in protein quality control.. Nature.

[R6] Brachmann CB, Davies A, Cost GJ, Caputo E, Li J, Hieter P, Boeke JD (1998). Designer deletion strains derived from Saccharomyces cerevisiae S288C: a useful set of strains and plasmids for PCR-mediated gene disruption and other applications.. Yeast.

[R7] Cabañas MJ, Vázquez D, Modolell J (1978). Dual interference of hygromycin B with ribosomal translocation and with aminoacyl-tRNA recognition.. Eur J Biochem.

[R8] Chen P, Johnson P, Sommer T, Jentsch S, Hochstrasser M (1993). Multiple ubiquitin-conjugating enzymes participate in the in vivo degradation of the yeast MAT alpha 2 repressor.. Cell.

[R9] Chuang SM, Madura K (2005). Saccharomyces cerevisiae Ub-conjugating enzyme Ubc4 binds the proteasome in the presence of translationally damaged proteins.. Genetics.

[R10] Crowder JJ, Geigges M, Gibson RT, Fults ES, Buchanan BW, Sachs N, Schink A, Kreft SG, Rubenstein EM (2015). Rkr1/Ltn1 Ubiquitin Ligase-mediated Degradation of Translationally Stalled Endoplasmic Reticulum Proteins.. J Biol Chem.

[R11] Daraghmi MM, Miller JM, Bailey CG, Doss EM, Kalinski AL, Smaldino PJ, Rubenstein EM (2023). Macro-ER-phagy receptors Atg39p and Atg40p confer resistance to aminoglycoside hygromycin B in S. cerevisiae.. MicroPubl Biol.

[R12] Doss EM, Moore JM, Harman BH, Doud EH, Rubenstein EM, Bernstein DA (2023). Characterization of endoplasmic reticulum-associated degradation in the human fungal pathogen Candida albicans.. PeerJ.

[R13] Flagg MP, Lam B, Lam DK, Le TM, Kao A, Slaiwa YI, Hampton RY (2023). Exploring the "misfolding problem" by systematic discovery and analysis of functional-but-degraded proteins.. Mol Biol Cell.

[R14] Guthrie C, Fink GR. 2004. Guide to Yeast Genetics and Molecular and Cell Biology. Methods in Enzymology 31. 2005781

[R15] Habeck G, Ebner FA, Shimada-Kreft H, Kreft SG (2015). The yeast ERAD-C ubiquitin ligase Doa10 recognizes an intramembrane degron.. J Cell Biol.

[R16] Huyer G, Piluek WF, Fansler Z, Kreft SG, Hochstrasser M, Brodsky JL, Michaelis S (2004). Distinct machinery is required in Saccharomyces cerevisiae for the endoplasmic reticulum-associated degradation of a multispanning membrane protein and a soluble luminal protein.. J Biol Chem.

[R17] Jaeger PA, Ornelas L, McElfresh C, Wong LR, Hampton RY, Ideker T (2018). Systematic Gene-to-Phenotype Arrays: A High-Throughput Technique for Molecular Phenotyping.. Mol Cell.

[R18] Kleiger G, Mayor T (2014). Perilous journey: a tour of the ubiquitin-proteasome system.. Trends Cell Biol.

[R19] Koegl M, Hoppe T, Schlenker S, Ulrich HD, Mayer TU, Jentsch S (1999). A novel ubiquitination factor, E4, is involved in multiubiquitin chain assembly.. Cell.

[R20] Liu C, van Dyk D, Choe V, Yan J, Majumder S, Costanzo M, Bao X, Boone C, Huo K, Winey M, Fisk H, Andrews B, Rao H (2011). Ubiquitin ligase Ufd2 is required for efficient degradation of Mps1 kinase.. J Biol Chem.

[R21] Liu C, van Dyk D, Xu P, Choe V, Pan H, Peng J, Andrews B, Rao H (2010). Ubiquitin chain elongation enzyme Ufd2 regulates a subset of Doa10 substrates.. J Biol Chem.

[R22] Matsumoto M, Yada M, Hatakeyama S, Ishimoto H, Tanimura T, Tsuji S, Kakizuka A, Kitagawa M, Nakayama KI (2004). Molecular clearance of ataxin-3 is regulated by a mammalian E4.. EMBO J.

[R23] Mehrtash AB, Hochstrasser M (2018). Ubiquitin-dependent protein degradation at the endoplasmic reticulum and nuclear envelope.. Semin Cell Dev Biol.

[R24] Müller L, Hoppe T (2024). UPS-dependent strategies of protein quality control degradation.. Trends Biochem Sci.

[R25] Nakatsukasa K, Huyer G, Michaelis S, Brodsky JL (2008). Dissecting the ER-associated degradation of a misfolded polytopic membrane protein.. Cell.

[R26] Niekamp JM, Evans MD, Scott AR, Smaldino PJ, Rubenstein EM (2019). *TOM1*
confers resistance to the aminoglycoside hygromycin B in
*Saccharomyces cerevisiae*
.. MicroPubl Biol.

[R27] Owutey SL, Procuniar KA, Akoto E, Davis JC, Vachon RM, O'Malley LF, Schneider HO, Smaldino PJ, True JD, Kalinski AL, Rubenstein EM (2024). Endoplasmic reticulum and inner nuclear membrane ubiquitin-conjugating enzymes Ubc6 and Ubc7 confer resistance to hygromycin B in Saccharomyces cerevisiae.. MicroPubl Biol.

[R28] Richly H, Rape M, Braun S, Rumpf S, Hoege C, Jentsch S (2005). A series of ubiquitin binding factors connects CDC48/p97 to substrate multiubiquitylation and proteasomal targeting.. Cell.

[R29] Rubenstein LD, Woodruff KA, Taylor AM, Olesen JB, Smaldino PJ, Rubenstein EM (2024). "Important Enough to Show the World": Using Authentic Research Opportunities and Micropublications to Build Students' Science Identities.. J Adv Acad.

[R30] Runnebohm AM, Evans MD, Richardson AE, Turk SM, Olesen JB, Smaldino PJ, Rubenstein EM (2020). Loss of protein quality control gene
*UBR1*
sensitizes
*Saccharomyces cerevisiae*
to the aminoglycoside hygromycin B.. Fine Focus.

[R31] Smith N, Adle DJ, Zhao M, Qin X, Kim H, Lee J (2016). Endoplasmic Reticulum-associated Degradation of Pca1p, a Polytopic Protein, via Interaction with the Proteasome at the Membrane.. J Biol Chem.

[R32] Tong AH, Evangelista M, Parsons AB, Xu H, Bader GD, Pagé N, Robinson M, Raghibizadeh S, Hogue CW, Bussey H, Andrews B, Tyers M, Boone C (2001). Systematic genetic analysis with ordered arrays of yeast deletion mutants.. Science.

[R33] Turk SM, Indovina CJ, Miller JM, Overton DL, Runnebohm AM, Orchard CJ, Tragesser-Tiña ME, Gosser SK, Doss EM, Richards KA, Irelan CB, Daraghmi MM, Bailey CG, Niekamp JM, Claypool KP, Engle SM, Buchanan BW, Woodruff KA, Olesen JB, Smaldino PJ, Rubenstein EM (2023). Lipid biosynthesis perturbation impairs endoplasmic reticulum-associated degradation.. J Biol Chem.

[R34] Turk SM, Indovina CJ, Miller JM, Overton DL, Runnebohm AM, Orchard CJ, Tragesser-Tiña ME, Gosser SK, Doss EM, Richards KA, Irelan CB, Daraghmi MM, Bailey CG, Niekamp JM, Claypool KP, Engle SM, Buchanan BW, Woodruff KA, Olesen JB, Smaldino PJ, Rubenstein EM (2023). Lipid biosynthesis perturbation impairs endoplasmic reticulum-associated degradation.. J Biol Chem.

[R35] Verma R, Oania RS, Kolawa NJ, Deshaies RJ (2013). Cdc48/p97 promotes degradation of aberrant nascent polypeptides bound to the ribosome.. Elife.

[R36] Watts SG, Crowder JJ, Coffey SZ, Rubenstein EM (2015). Growth-based determination and biochemical confirmation of genetic requirements for protein degradation in Saccharomyces cerevisiae.. J Vis Exp.

[R37] Wong ED, Miyasato SR, Aleksander S, Karra K, Nash RS, Skrzypek MS, Weng S, Engel SR, Cherry JM (2023). Saccharomyces genome database update: server architecture, pan-genome nomenclature, and external resources.. Genetics.

[R38] Woodruff KA, Richards KA, Evans MD, Scott AR, Voas BM, Irelan CB, Olesen JB, Smaldino PJ, Rubenstein EM (2021). Inner Nuclear Membrane Asi Ubiquitin Ligase Catalytic Subunits Asi1p and Asi3p, but not Asi2p, confer resistance to aminoglycoside hygromycin B in
*Saccharomyces cerevisiae*
.. MicroPubl Biol.

[R39] Wu H, Pomeroy SL, Ferreira M, Teider N, Mariani J, Nakayama KI, Hatakeyama S, Tron VA, Saltibus LF, Spyracopoulos L, Leng RP (2011). UBE4B promotes Hdm2-mediated degradation of the tumor suppressor p53.. Nat Med.

